# Editorial: Understanding Immunobiology Through the Specificity of NF-κB

**DOI:** 10.3389/fimmu.2020.00059

**Published:** 2020-01-30

**Authors:** Myong-Hee Sung, Sergi Regot

**Affiliations:** ^1^Laboratory of Molecular Biology and Immunology, National Institute on Aging, National Institutes of Health, Baltimore, MD, United States; ^2^Department of Molecular Biology and Genetics, Johns Hopkins University, Baltimore, MD, United States

**Keywords:** NF-kappaB (NF-κB), microscopy, systems biology, quantitative methodologies, high throughput profiling, mathematical modeling

Since its discovery more than 30 years ago, the significance of NF-κB transcription factors has penetrated virtually all areas of biomedical research. While it was originally found from immune cells, these evolutionarily conserved proteins are expressed in most cell types. Dysregulation of NF-κB has been observed in many devastating diseases such as cancer, autoimmunity, and neurodegenerative diseases. NF-κB biology has been intensely studied over the years, and numerous regulatory mechanisms and target genes have been identified. Briefly, NF-κB exists as hetero/homo-dimers whose subunits share a Rel homology domain which mediates the dimerization. Even though NF-κB constitutively shuttles between the cytoplasm and the nucleus, NF-κB dimers are efficiently retained in the cytoplasm by the inhibitor of NF-κB (IκB) proteins in a resting state. Upon stimulation, the ubiquitin-proteasome-mediated degradation of IκBs results in nuclear accumulation of NF-κB, where these transcription factor proteins scan the tissue-specific epigenome and bind to κB motifs within accessible chromatin in a matter of minutes, to regulate tissue-specific gene expression programs. At individual promoters or enhancers, NF-κB collaborates with or antagonizes other transcriptional regulators which brings about distinct regulatory outcomes. The magnitude and duration of NF-κB action are governed by multiple negative and positive feedback regulators.

In light of its ubiquitous expression and the conserved core regulatory module described above, the function of NF-κB seems remarkably specific to the distinct signals from the microenvironment or intracellular stress. Despite the great advances in the field, we still lack the knowledge about detailed workings of transcriptional regulation by NF-κB and the functional relevance. For example, recent technological advances, some of which discussed in this Research Topic, have shown unexpected complexity regarding the temporal and spatial regulation of NF-κB activity. However, the role of NF-κB dynamics in fine-tuning epigenetic and transcriptional programs remains poorly understood. The remaining frontiers of investigation into NF-κB are likely to hold the key to the information that we need to control this transcriptional regulator for therapeutic gains in several pathological settings. Here, we have collected reviews and research reports from some of the investigators who have shaped our current knowledge and continue to shed light on NF-κB biology.

Brignall et al. contributed a Review which presents an insightful in-depth discussion of a range of subtopics, from NF-κB dimer specificity to genomic binding site selection. These are critical areas that will need further elucidation for a quantitative understanding of NF-κB functioning as a transcription factor.

In a Review, Jeknić et al. summarize technical approaches that address an aspect of NF-κB regulatory mechanisms which received a relatively late attention: how the temporal patterns of NF-κB signaling dynamics are used to encode functional information and how they are decoded by individual cells. They discuss the challenges of generating informative single-cell measurements and survey currently available analysis and engineering tools, as well as recent improvements in throughput and information content of various assay platforms.

Nelson and Nelson's Mini Review highlights the importance of single cell analysis in examining host-pathogen interactions to account for the different infection status in individual cells. The article explains key findings from the pioneering studies of cellular responses to intact microbes which allow the investigation of not only a full host defense response but also how NF-κB is modulated by invading pathogens for their survival.

Tissue-specificity of NF-κB function is influenced by the repertoire of intracellular factors interacting with the regulators of NF-κB within a given cellular context. Macrophages are important effectors of innate immunity, and recent evidence suggests that findings about NF-κB signaling in non-immune cells such as fibroblasts may not apply to this cell type. Macrophage-specific NF-κB signaling mechanisms is the focus of a Review by Dorrington and Fraser. The central nervous system (CNS) has become an exciting context for NF-κB in recent years, since the brain represents an organ where NF-κB can have strikingly distinct functions depending on the cellular context such as neurons or glia. Moreover, microglia, the resident immune cells in the brain, emerge as the relevant cells for manifesting the phenotypes of SNPs associated with neurodegenerative diseases such as Alzheimer's disease. The Review by Dresselhaus and Meffert is a timely exposition of NF-κB neurobiology in various CNS cell components and their roles in several neurodegenerative diseases.

A Review by Adelaja and Hoffmann summarizes data on how NF-κB signaling is modulated by crosstalk mechanisms between pathways that are downstream of TLR ligands, IL-1, TNF-α, lymphotoxins, and interferons.

The Research Topic also includes three primary research articles from studies using diverse tools. As discussed extensively in the Review by Brignall et al., the dimer specificity of NF-κB proteins and its functional relevance are poorly understood. Martin et al. analyzed the dimerization status of RelA subunit of NF-κB using a quantitative live cell microscopy technique termed Number and Brightness. The result suggests that a higher than expected proportion of NF-κB dimers exist as RelA:RelA homodimers.

Chatterjee et al. show that surprises can still be found regarding biochemical networks of NF-κB. They describe an unexpected role of p100 in regulating the canonical NF-κB pathway downstream of TNF-α.

Mitchell et al. took a mathematical modeling approach and present a simplifying tissue-level NF-κB activity “calculator,” showing that the activation status of a pathway as complex as NF-κB can be projected onto a simple measure with useful predictive features. Their calculator was able to dissect distinct macrophage mechanisms of type I and II interferons in amplifying NF-κB activity.

While the Research Topic showcases some important recent fruits and open problems in the field, we acknowledge that this collection does not encompass all the significant lines of research that deserve our attention. Exciting technological innovations are enabling us to address previously intractable questions about how the NF-κB system is used for interpreting danger- or stress-associated signals with a robust functional specificity. At the same time, this collection also reminds us that we still have a long way to go toward understanding the biology of NF-κB in the immune system and beyond.

## Author Contributions

M-HS and SR wrote the manuscript.

### Conflict of Interest

The authors declare that the research was conducted in the absence of any commercial or financial relationships that could be construed as a potential conflict of interest.

